# Antimicrobial Resistance Profiles of *Staphylococcus aureus* Isolated from Meat Carcasses and Bovine Milk in Abattoirs and Dairy Farms of the Eastern Cape, South Africa

**DOI:** 10.3390/ijerph15102223

**Published:** 2018-10-11

**Authors:** Abongile Pekana, Ezekiel Green

**Affiliations:** 1 Department of Biochemistry and Microbiology, University of Fort Hare, Alice 5700, South Africa; 200600011@ufh.ac.za; 2 Department of Biotechnology and Food Technology, University of Johannesburg, Doornfontein 2028, South Africa

**Keywords:** *Staphylococcus aureus*, meat, raw milk, antibiotics, antibiotic resistance genes

## Abstract

*Background*: *Staphylococcus aureus* (*S. aureus*) occasionally threatens the life of the host as a persistent pathogen even though it is normal flora of humans and animals. We characterized drug resistance in *S. aureus* isolated from animal carcasses and milk samples from the abattoirs and dairy farms in the Eastern Cape Province. *Methods*: A total of 1000 meat swab samples and 200 raw milk samples were collected from selected abattoirs and dairy farms. *S. aureus* was isolated and positively identified using biochemical tests and confirmed by molecular methods. An antibiotic susceptibility test was performed on all isolates for 14 antibiotics and correspondent genes were detected. *Results*: Of the 1200 samples collected, 134 (11.2%) samples were positive for *S. aureus*. Resistance ranged from 71.6% for penicillin G to 39.2% for tetracycline. A resistance gene (*bla*Z) was detected in 13 (14.9%), while *msr*A was found in 31 (52.5%) of *S. aureus* isolates. *Conclusions*: The present result shows the potential dissemination of multidrug-resistant *S. aureus* strains in the dairy farms and abattoirs in the Eastern Cape. Therefore, this implies that the organism may rapidly spread through food and pose serious public health risk.

## 1. Introduction

Staphylococci asymptomatically colonizes the skin and mucous membranes in the nostrils of humans and animals [1,2,3,4,5]. This is an important outcome, bearing in mind the fact that nasal carriage of *Staphylococcus aureus* has been associated with subsequent infection [5]. Several studies have reported the identification of coagulase-positive and coagulase-negative species in warm-blooded animals [2,3,6,7,8,9]. Carriers are therefore an important source infection spread in communities. *S. aureus* (*Staphylococcus aureus*) causes diseases in humans and animals which include toxic syndrome and staphylococcal food poisoning (SFP) [10,11,12,13]. The work of Hatakka et al. [14] has revealed that *S. aureus* in meat is a result of improper hygienic practices during handling by the slaughter personnel during meat production.

South African studies have reported that a high percentage of the population largely depends on beef and pork meat as a protein [15,16]. Additionally, some researchers have demonstrated that infections with antibiotic resistant strains are caused by foods contaminated with antibiotic resistant bacteria [17,18,19] making them an ideal vehicle for transmission of antibiotic resistance.

Studies have reported that prolonged use and misuse of antimicrobial agents in agriculture, stock farming and in treatment of human diseases have resulted in rapid resistance of many bacteria to several antibiotics of different classes [20,21,22]. The development of antibiotic resistance has been observed for a variety of antimicrobial agents which include aminoglycosides, macrolides, glycopeptides, fluoroquinolones and tetracyclines [23]. Many antibiotic resistance genes play a role in *S. aureus* resistance and these include macrolide resistance encoded by the *erm* gene, *aphA3* and *sat* genes for kanamycin and streptomycin resistance and *accA-aphD* and *tet* genes for gentamicin, tobramycin and tetracycline resistance [24,25].

There is paucity of information on the molecular characterization of *S. aureus* in most developing countries [23,26] especially in the Eastern Cape province of South Africa. Better understanding of *S. aureus* antibiotic susceptibility profiles and molecular characterization of genes causing resistance are of paramount importance for initiating effective control measures and reducing staphylococcal infections [23,26]. The aim of the study was to identify and characterize antibiotic resistance susceptibility patterns including antibiotic resistance genes in *S. aureus* strains isolated from selected dairy farms and abattoirs in the Eastern Cape Province, South Africa.

## 2. Materials and Methods 

A total of two hundred milk samples were collected from cows with subclinical and clinical mastitis cases over 6 months at Dairy Farm A (100 samples) and Dairy Farm B (100 samples). Milk samples were collected using the method of Caine et al. [27]. Briefly, in each milking station there is a small collection bottle with a small hole that is opened and closed using a tap. The bottles were properly washed and used for sampling another cows’ milk. All the milk samples were stored on ice and transported to the Biochemistry and Microbiology Laboratory for analysis. 

A total of 1000 meat swab samples were collected from cow carcasses, pig carcasses and sheep carcasses in four selected abattoirs according to the method of Pearce and Bolton [18]. Permission to collect swab samples was obtained from abattoir managers. Samples were collected from the available animal carcasses during a period of 10 months (August 2015 to May 2016). Samples were collected using a sterile swab rinsing kit containing 10 mL isotonic buffer rinse solution, after the gastrointestinal tract was removed. A 100 cm^2^ sterile disposable plastic template (Analytical Diagnostics, USA) was used to mark the area for swabbing. A total of 500 meat swab samples were collected from cow carcasses, 300 meat swab samples from sheep carcasses and 200 meat swab samples from pig carcasses. Each animal carcass was sampled in four areas which included rump, flank, brisket, and neck, and isolates from those four areas were counted consecutively. The meat swab samples were then stored on ice and transported to the Biochemistry and Microbiology Laboratory for analysis.

### 2.1. Isolation of S. aureus Milk and Meat Samples

Ten microliters of each milk sample were inoculated onto Baird Parker Agar (Oxoid; ThermoScientific, England, UK) and incubated at 37 °C for 24–48 h. Meat swab samples were also inoculated onto the same culture media (in-house method) and incubated for the same period. Presumptive grey-black colonies surrounded by opaque halo of precipitation on Baird Parker agar were regarded as presumptive *S. aureus* isolates and were subjected to biochemical identification. 

### 2.2. Biochemical Identification and DNA Extraction

Gram-staining, catalase test and oxidase test were performed according to the method of Health Protection Agency [28,29,30] for biochemical identification of the organism. DNA extraction was performed based on the procedure of Maugeri et al. [31]. Briefly, a loop full 24-h culture of *S. aureus* colonies grown onto Nutrient Agar plates were suspended into 200 µL of sterile nuclease free water and vortexed for 2 min using MS2 Minishaker (Digisystem Laboratory instruments Inc., New Taipei City, Taiwan) and the cells were lysed using a heat Dri-Block DB.2A (Technc, Johannesburg, South Africa) for 15 min at 100 °C. The pellet was removed by centrifugation at 10,000 rpm for 5 min using a MiniSpin microcentrifuge (ThermoFisher Scientific, Waltham, MA, USA) kept at 4 °C. The supernatant was transferred in new Eppendorf tubes and used for PCR reactions.

### 2.3. Bacterial Identification

#### The *nuc* Gene Amplification

The isolates were confirmed by PCR amplification of the *nuc* gene encoding the thermonuclease enzymes with the oligonucleotide primers shown in Table 1. The total PCR reaction volume of 25 μL containing 12 μL of master mix (Kapa Biosystems, Johannesburg, South Africa) (containing, DNA Taq polymerase, dNTPs, MgCl_2_ and PCR buffer), 5 μL DNA template, 1 μL of the forward and reverse primers, and 6 μL of nuclease free water was used. Polymerase chain reaction (PCR) was performed using MyCycler^TM^ (Biorad, Cape Town, South Africa). A total of 35 PCR cycles were run under the following conditions: DNA denaturation at 94 °C for 1 min, primer annealing at 55 °C for 0.5 min, and DNA extension at 72 °C for 1.5 min. The final cycle was at 72 °C for 5 min. The PCR products were stored at 4 °C until they were collected. Amplicons were resolved on 1.5% agarose gel containing 5 μL Ethidium bromide in 1× TAE buffer pH 8.0 for 1 h at 100 V before being visualized and captured under Alliance 4.7 transilluminator (UVITEC Limited, Cambridge, UK).

### 2.4. Antibiotic Susceptibility Testing

A disk diffusion antibiotic susceptibility test was conducted according to the Clinical and Laboratory Standards Institute [32]. Bacterial suspensions were prepared in 2.5 mL of Mueller-Hinton broth and the turbidity was adjusted to meet 0.5 McFarland turbidity standards (~1.5 × 10^8^ cfu/mL). The isolates were inoculated onto a Mueller-Hinton Agar plate and tested against a panel of fourteen antibiotics (Table 2) and results were interpreted according to the Clinical and Laboratory Standards Institute [32]. *Staphylococcus aureus* ATCC 25923 was used as a positive control. A dendrogram was generated by unweighted pairwise grouping with mathematical averaging (UPGMA). Isolates were considered as belonging to a common cluster when the resistance pattern differed by ≤5 antibiotics.

#### Detection of Antibiotic Resistance Genes of *Staphylococcus aureus*

The confirmed *S. aureus* isolates were screened for antibiotic resistance genes using the oligonucleotide primers and PCR conditions listed in Table 1. The total reaction volume of 25 μL containing 12 μL of master mix (Kapa Biosystems, Johannesburg, South Africa) (containing, DNA Taq polymerase, dNTPs, MgCl_2_ and PCR buffer), 5 μL DNA template, 1 μL of the forward and reverse primers (10 ng), and 6 μL of nuclease free water was used for amplification. Polymerase chain reaction (PCR) was performed using MyCycler^TM^ (Biorad, Cape Town, South Africa). The amplified products were separated on 1.5% agarose gel containing 5 μL Ethidium bromide in 1× TAE buffer pH 8.0 for 1 h at 100 V before being visualized and photographed under Alliance 4.7 transilluminator (UVITEC Limited, Cambridge, UK).

## 3. Results

### 3.1. Study Population

From 1100 meat and milk samples, a total of 134 samples were positive for *S. aureus* by culture, biochemical tests and molecular confirmed by polymerase chain targeting the *nuc* gene. There were 102/500 (20.4%) isolates from beef samples, 10/300 (3.3%) from sheep samples, 14/100 (14%) from pork samples were 8/200 (4%) from milk samples. All of the 134 *S. aureus* revealed a 255 base pair size in an agarose gel electrophoresis.

### 3.2. Antimicrobial Resistance Screening of S. aureus Isolates

The resistance profiles of isolated *S. aureus* are shown in Table 2. Sheep and pork isolates were resistant to one to five antimicrobial agents. Multidrug resistance (MDR) among all *S. aureus* isolates was noted in penicillin G, oxacillin, clindamycin, erythromycin and tetracycline (Table 3). 

Table 2 shows *Staphylococcus aureus* isolates resistant to penicillin G per sampling site. Resistance from isolates obtained from rump ranged from 40–62.9%, flank ranged from 60–100%, brisket from 50–100% and the neck from 20–100% from pork, sheep and beef isolates. A similar antibiotic resistance pattern was also observed for oxacillin, where isolates from rump ranged from 40–75%, flank 50–100%, brisket 50–100%, and neck 20–100% while resistance to tetracycline was observed in isolates from rump ranging from 40–75%, isolates from flank with percentages ranging from 45–50%, brisket 25–50% and neck 20–50%.

Multidrug-resistance was observed in 18 isolates from different farms and abattoirs. The pattern of resistance ranged from two antibiotics to nine antibiotics (Table 3).

**Figure 1:**
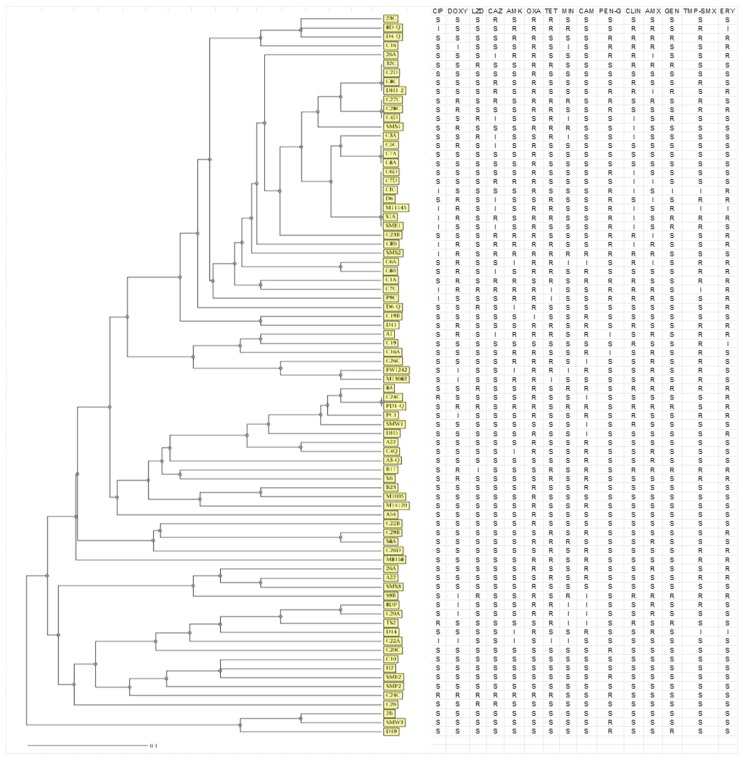
Dendrogram representing the similarity grouping of *Staphylococcus aureus* clusters based on their susceptibility profiles. The MIRU-VNTR*plus* service (www.miru-vntrplus.org) was used to compare isolates (resistance coded as 2, susceptible as 1 and intermediate as 1). The differences in susceptibility profiles were used to estimate the distance. The strains were encoded and shown by numbers and letters highlighted in yellow. (CIP: Ciprofloxacin, Doxy: Doxycycline, LZD: Linezoid, CAZ: Ceftazidime, AMK: Amikacin, OXA: Oxacillin, TET: Tetracycline, MIN: Minocycline, CAM: Chloramphenicol, PEN-G: Penicillin-G, CLI: Clindamycin, AMX: Amoxicillin, GEN: Gentamicin, TMP-SMX: Trimethoprim-sulfamethoxazole, ERY: Erythromycin.)

Among the 87 isolates resistant to different antibiotics, 65 different patterns were observed with 5 clusters. The patterns presented between 2 and 7 isolates. The remaining patterns were represented by single isolates (Figure 1).

#### Detection of Resistance Genes

Resistant genes detected ranged from 3.8% for *tet*K in sheep and 79.3% for *tet*M in beef. Sheep, pork and milk showed no detection of *mec*A, *tetM*, *ant (4′)-Ia* and *aph (3′)-1-IIIa* (Table 4). All other genes tested were not detected.

## 4. Discussion

Antibiotic use drives the evolution of antibiotic resistance [33]. Our task is to preserve the effectiveness of existing antibiotics by minimizing the emergence and spread of multidrug resistant microorganisms to maximize the time until existing antibiotics become ineffective. Over the past years, the dissemination of antimicrobial resistance (AR) in bacteria, including staphylococci has increased and poses public health risks. This is best narrated by the multidrug resistant *S. aureus* strains that cause infections that are difficult to treat [38]. In this study the most *S. aureus* isolates were observed in beef samples and beef isolates showed resistance to several antibiotics including penicillin G (71.6%), oxacillin (66.7%), clindamycin (52.9%), erythromycin (48%) and tetracycline (39.2%) respectively. Sheep and pork samples were relatively resistant to one to five antimicrobial agents. The work conducted by Yang et al. [39] has reported similar levels of resistance of *S. aureus* isolates to penicillin, erythromycin and tetracycline [40,41]. Andreotti and Nicodeno [42] have revealed that the resistance of *S. aureus* to penicillin ranges from 20% to 100%, whilst the percentage of resistance to other antibiotics was relatively lower. Most of the isolates in our study showed high sensitivity to several antibiotics including ciprofloxacin, chloramphenicol, clindamycin, linezolid and trimethoprim-sulfamethoxazole. This implies that such antibiotics can be used to treat infections caused by *S. aureus*. In our study, 65 different patterns were observed with 5 clusters among which, isolate Dh1-2 and 32C are from two different abattoirs while M11145 and SME1 were isolated from different farms isolated from milk. The patterns presented between 2 and 7 isolates. The remaining patterns were represented by single isolates. However, this is not a true reflection of clusters as only one aspect was used and no typing methods such as Pulse Field Gel Electrophoresis (PFGE), ribotyping and phagotyping were used.

In this study 82 (67%) isolates showed phenotypic resistance to methicillin, however only one methicillin-resistant *Staphylococcus aureus* (MRSA) gene was detected using molecular methods. All the other isolates that showed resistance to oxacillin could have a *mec*C gene, which was not investigated in our study. Further studies need to be carried out to ascertain the involvement of the *mecC* gene in resistance of these isolates to oxacillin. A study performed by Diederen et al. [43] in the Netherlands has demonstrated that 2.5% of pork and beef samples harbored MRSA isolates. The research performed by Fessler and co-workers [44] has shown that MRSA were resistant to oxacillin, and 62.5% exhibited multidrug resistance. Similarly, the study of Hanson et al. [44] advocated that MRSA isolated from pork, beef, chicken and turkey were resistant to oxacillin and several other antibiotics. There were 87 (69%) *S. aureus* isolates that were phenotypically resistant to penicillin G and only 13 (14.9%) isolates were found to express the *bla*Z gene in this study. Disagreement can occur between phenotypic and genotypic resistance due to incomplete understanding of the genotypic basis of phenotypic resistance, flaws with the phenotypic or molecular (e.g., PCR) methods currently used to detect resistance. It may also be because the phenotypic resistance has been caused by point mutations, biofilm formation or antibiotic tolerance [36,45]. Different studies demonstrate the presence of other virulence factors that must be taken in consideration for a comprehensive evaluation of the risk. MRSA and MSSA can be considered parameters for the valuation of dedicated food chains: Poultry meats [46] and sheep (ovine food-chain) [47]. Specific genomic features, as *pvl* or *mecC,* were reported recently as emergent virulence factors in livestock in different papers [48,49].

Of the 82 (67%) *S. aureus* isolates that were phenotypically resistant to oxacillin, only one isolate (1.2%) was found to possess the *mec*A gene. This small proportion of isolates that showed amplification of the *mec*A gene compared to phenotypic resistance to oxacillin was not surprising. Oxacillin has been proposed as an alternative antibiotic for testing susceptibility/resistance to methicillin and to all β-lactams [50]. This could explain why all oxacillin-resistant isolates were not carrying the *mec*A gene, because they were showing resistance to β-lactams. Phenotypic resistance witnessed to oxacillin in this study could have been attained through other mechanisms, including the reduction in membrane permeability to β-lactam antibiotics.

Binding of tetracycline antibiotics to the 30S ribosomal subunit prevents association of aminoacyl-tRNA with its acceptor site, thereby inhibiting protein synthesis [51]. *S. aureus* uses two mechanisms of tetracycline resistance; active efflux via *tet*A (*K*) and *tet*A(*L*) and ribosomal protection via *tet*A(*M*) [51]. Tetracycline efflux in *S. aureus* strains is mediated by *tet*A(*K*), which is commonly carried by plasmid pT181. Integration of this plasmid into Type III *SCCmec* makes this kind of resistance to be named chromosomally encoded resistance. Resistance to tetracycline can also be mediated by mutations that cause increased expression of various chromosomally encoded efflux pumps, such as Tet38 [52,53]. In this study, a high detection rate was observed in *tetM* 29 (46.7%) while *tetK* detection rate was 26 (41.9%) for tetracycline resistant genes. Seven (11.3%) isolates contained both *tet*K and *tet*M gene. 

The *erm*(C) determinant was found in 2 isolates (3.4%) while no *erm*A and *erm*B were detected. These results are different from the results of Cetin et al. [54] who showed that *erm*A was the most prevalent phenotype among *S. aureus*. Macrolide antibiotic (including azithromycin, clarithromycin, erythromycin), resistance in *S. aureus* may be due to an active drug efflux mechanism encoded by *msr*A and *msr*B (conferring resistance to macrolides and type B streptogramins). In our study, *msr*A determinant was detected in 31 (52.5%) isolates. It is likely that other erythromycin resistance genes such as *msr*A, *Ere* A–B or *mef,* which we did not include in our study, might be present among these isolates and account for the remainder of the isolates showing phenotypic resistance to erythromycin.

Aminoglycosides antibiotics play an important role in the treatment of staphylococcal infections [55]. Aminoglycosides inactivate antibiotics using aminoglycoside-modifying enzymes (AMEs) that are encoded by genetic elements [56,57]. The *aac (6′)-Ie + aph (2″)*, *ant (4′)-Ia*, *aph (3′)-IIIa*, and *ant (6)-Ia* genes that encode aminoglycoside-6′-*N*-acetyltransferase/2″-*O*-phosphoryltransferase, aminoglycoside-4′-*O*-nucleotidyltransferase I, aminoglyoside-3′-*O*-phosphoryltransferase III, and streptomycin modifying enzyme, respectively, are the most important genes in this regard. Resistance to gentamicin, kanamycin, and tobramycin in staphylococci is mediated by a bi-functional enzyme displaying AAC (6′) and APH (2″) activity [58]. In our study, we found 15 *S. aureus* isolates which were phenotypically resistant to gentamicin, however, only 5 (33.3%) isolates were found to express one of these gentamicin resistance genes. Antibiotic resistance can be classified into three main categories: Intrinsic, adaptive, and acquired resistance [59]. We speculate that intrinsic antibiotic resistance, being the naturally low permeability of the bacterial cell wall, which limits uptake of many antibiotics including aminoglycosides is responsible for the other 10 isolates that did not show amplicons of the investigated target genes. We did not encounter any organism that was resistant to all tested antibiotics, implying that other tested antibiotics could still be used to inhibit the growth *S. aureus* in the from those four abattoirs and two farms investigated.

Although resistance to antibiotics were present, a characteristic typically reported in pork [18], the high proportion of multidrug resistance demonstrates how common *S. aureus* multidrug resistance is in the studied dairy farms and abattoirs. This development might be linked to numerous factors. Most dairy farmers in the region are not aware of the risks of *S. aureus* colonization to milk production and human health. Hygiene measures were not maintained before and after milking or slaughtering, which may expedite the presence of *S. aureus* on the skin of the udder and its access to the mammary gland and the different sites sampled. The importance of hygiene measures is highlighted in this study. 

## 5. Conclusions

Antibiotic misuse has determined resistance and *S. aureus* is widely implicated in antibiotic resistance transfer. However, better supervision and the proper application of hygienic practices in the transformation of raw materials of animal origin can reduce the spread of antibiotic resistance *S. aureus*. The distribution of antibiotic resistance is high in the Eastern Cape Province as compared to other areas where culling of infected animals is done. Usage of molecules in veterinary medicine and animal husbandry that are identical as, or closely associated to, antibiotics used in human medicine should be greatly discouraged.

## Figures and Tables

**Table 1:** Oligonucleotide sequences used for polymerase chain reaction in the identification of *Staphylococcus aureus* and detection of antibiotic resistance genes.

Target Gene	Oligonucleotide Sequence (5′-3′)	Amplicon Size (bp)	PCR Conditions	Reference
*nuc*	GCG ATT GAT GGT GAT ACG GTT CCA AGC CTT GAC GAA CTA AAG C	255	PCR was performed under the following conditions: One cycle of 95 °C for 5 min, 35 cycles of 95 °C for 30 s, 55 °C for 30 s, and 72 °C for 1 min, 1 cycle of 72 °C for 10 min.	[33]
*mec*A	AAA ATC GAT GGT AAA GGT TGG C AGT TCT GCA GTA CCG GAT TTG C	533	Amplification was carried out using 40 cycles of amplification at 94 °C for 30 s, 55 °C for 30 s, and 72 °C for 1 min; this reaction was followed by 5 min of an additional extension at 72 °C.	[6]
*bla*Z	ACT TCA ACA CCT GCT GCT TTC TGA CCA CTT TTA TCA GCA ACC	173	PCR mixture was subjected to 35 cycles of amplification. The conditions for each cycle were: Denaturation for 1 min at 94 °C, annealing for 1 min at 54 °C, and primer extension for 1 min at 72 °C. Finally, the reaction was incubated at 72 °C for 10 min.	[34]
*acc(6)-aph(2)*	TTG GGA AGA TGA AGT TTT TAG A CCT TTA CTC CAA TAA TTT GGC T	174	95 °C for 5 min was followed by 30 cycles of denaturation at 95 °C for 60 s, and then annealed at 54 °C for 2 min, and after that extended at 72 °C for 2 min. Finally, an additional extension was achieved for 5 min at 72 °C.	[35]
*aph (3’)-1-IIIa*	AAA TAC CGC TGC GTA CAT ACT CTT CCG AGC AA	242	95 °C for 5 min was followed by 30 cycles of denaturation at 95 °C for 30 s, and then annealed at 54 °C for 1 min, and after that extended at 72 °C for 2 min. Finally, an additional extension was achieved for 5 min at 72 °C.	[36]
*ant (4’)-Ia*	AAT CGG TAG AAG CCC AA GCA CCT GCC ATT GCT A	135	95 °C for 3 min was followed by 30 cycles of denaturation at 95 °C for 60 s, and then annealed at 54 °C for 2 min, and after that extended at 72 °C for 2 min. Finally, an additional extension was achieved for 5 min at 72 °C.	[36]
*erm*A	TAT CTT ATC GTT GAG AAG GGA TT CTA CAC TTG GCT TAG GAT GAA A	139	95 °C for 5 min was followed by 30 cycles of denaturation at 95 °C for 30 s, and then annealed at 54 °C for 1 min, and after that extended at 72 °C for 2 min. Finally, an additional extension was achieved for 5 min at 72 °C.	[35]
*erm*B	CTA TCT GAT TGT TGA AGA AGG ATT GTT TAC TCT TGG TTT AGG ATG AAA	142	95 °C for 5 min was followed by 30 cycles of denaturation at 95 °C for 30 s, and then annealed at 54 °C for 1 min, and after that extended at 72 °C for 2 min. Finally, an additional extension was achieved for 5 min at 72 °C.	[37]
*erm*C	CTT GTT GAT CAC GAT AAT TTC C ATC TTT TAG CAA ACC CGT ATT C	190	95 °C for 5 min was followed by 30 cycles of denaturation at 95 °C for 30 s, and then annealed at 54 °C for 1 min, and after that extended at 72 °C for 2 min. Finally, an additional extension was achieved for 5 min at 72 °C.	[35]
*msr*A	TCC AAT CAT TGC ACA AAA TC AAT TCC CTC TAT TTG GTG GT	163	94 °C for 5 min, 30 cycles of 94 °C for 45 s, 50 °C for 60 s, 72 °C for 60 s, final extension at 72 °C for 10 min.	[35]
*tet*K	GTA GCG ACA ATA GGT AAT AGT GTA GTG ACA ATA AAC CTC CTA	360	Initial denaturation at 94 °C for 3 min, 25 cycles of 94 °C for 1 min, annealing at 55 °C for 1 min, and extension at 72 °C for 1 min. The final extension was carried out at 72 °C for 10 min.	[37]
*tet*M	AGT GGA GCG ATT ACA GAA CAT ATG TCC TGG CGT GTC TA	158	Initial denaturation at 94 °C for 3 min, 25 cycles of 94 °C for 1 min, annealing at 55 °C for 1 min, and extension at 72 °C for 1 min. The final extension was carried out at 72 °C for 10 min.	[37]

**Table 2:** Antibiotic resistance of *Staphylococcus aureus* isolates in beef, sheep and pork samples against 14 different antibiotics.

Antimicrobial Resistance					
Antibiotics	Rump *np*/*n* (%)	Flank *np*/*n* (%)	Brisket *np*/*n* (%)	Neck *np*/*n* (%)	Total *np*/*n* (%)
Penicillin G					
Beef	22/35 (62.9)	12/20 (60)	23/28 (82.1)	16/19 (84.2)	73/102 (71.6)
Sheep	2/4 (50)	2/2 (100)	1/2 (50)	2/2 (100)	7/10 (70)
Pork	2/5 (40)	0/0 (0)	4/4 (100)	1/5 (20)	7/14 (50)
Oxacillin/Methicillin					
Beef	23/35 (65.7)	10/20 (50)	16/28 (57.1)	19/19 (100)	68/102 (66.7)
Sheep	3/4 (75)	2/2 (100)	1/2 (50)	1/2 (50)	7/10 (70)
Pork	2/5 (40)	0/0 (0)	4/4 (100)	1/5 (20)	7/14 (50)
Tetracycline					
Beef	14/35 (40)	9/20 (45)	11/28 (39.3)	6/19 (31.6)	40/102 (39.2)
Sheep	3/4 (75)	1/2 (50)	1/2 (50)	1/2 (50)	6/10 (60)
Pork	0/5 (0)	0/0 (0)	1/4 (25)	1/5 (20)	2/14 (14.3)
Doxycycline					
Beef	9/35 (25.7)	5/20 (25)	6/28 (21.4)	6/19 (31.6)	26/102 (25.5)
Sheep	2/4 (50)	0/2 (0)	0/2 (0)	0/2 (0)	2/10 (20)
Pork	1/5 (20)	0/0 (0)	0/4 (0)	1/5 (20)	2/14 (14.3)
Minocycline					
Beef	4/35 (11.4)	3/20 (15)	2/28 (7.1)	4/19 (21.1)	13/102 (12.7)
Sheep	1/4 (25)	0/2 (0)	0/2 (0)	0/2 (0)	1/10 (10)
Pork	1/5 (20)	0/0 (0)	0/4 (0)	0/5 (0)	1/14 (7.1)
Erythromycin					
Beef	15/35 (42.9)	6/20 (30)	15/28 (53.6)	13/19 (68.4)	49/102 (48)
Sheep	2/4 (50)	2/2 (100)	1/2 (50)	1/2 (50)	6/10 (60)
Pork	1/5 (20)	0/0 (0)	2/4 (50)	1/5 (20)	4/14 (28.6)
Amikacin					
Beef	9/35 (25.7)	2/20 (10)	5/28 (17.9)	6/19 (31.6)	22/102 (21.6)
Sheep	1/4 (25)	0/2 (0)	0/2 (0)	1/2 (50)	2/10 (20)
Pork	0/5 (0)	0/0 (0)	0/4 (0)	0/5 (0)	0/14 (0)
Gentamicin					
Beef	1/35 (2.9)	2/20 (10)	2/28 (7.1)	5/19 (26.3)	10/102 (9.8)
Sheep	2/4 (50)	1/2 (50)	0/2 (0)	2/2 (100)	5/10 (50)
Pork	0/5 (0)	0/0 (0)	0/4 (0)	0/5 (0)	0/14 (0)
Ciprofloxacin					
Beef	2/35 (5.7)	0/20 (0)	2/28 (7.1)	2/19 (10.5)	6/102 (5.9)
Sheep	0/4 (0)	0/2 (0)	0/2 (0)	1/2 (50)	1/10 (10)
Pork	0/5 (0)	0/0 (0)	0/4 (0)	0/5 (0)	0/14 (0)
Clindamycin					
Beef	20/35 (57.1)	8/20 (40)	13/28 (46.4)	13/19 (68.4)	54/102 (52.9)
Sheep	1/4 (25)	1/2 (50)	2/2 (100)	2/2 (100)	6/10 (60)
Pork	1/5 (20)	0/0 (0)	2/4 (50)	0/5 (0)	8/14 (57.1)
Chloramphenicol					
Beef	0/35 (0)	3/20 (15)	0/28 (0)	0/19 (0)	3/110 (2.7)
Sheep	0/4 (0)	0/2 (0)	0/2 (0)	0/2 (0)	0/10 (0)
Pork	0/5 (0)	0/0 (0)	0/4 (0)	1/5 (20)	1/14 (7.1)
Trimethoprim/sulfomethoxazole					
Beef	9/35 (25.7)	5/20 (25)	8/28 (28.6)	3/19 (15.8)	25/102 (24.5)
Sheep	1/4 (25)	0/2 (0)	0/2 (0)	0/2 (0)	1/10 (10)
Pork	1/5 (20)	0/0 (0)	1/4 (25)	0/5 (0)	2/14 (14.3)
Ceftaroline					
Beef	5/35 (14.3)	5/20 (25)	9/28 (32.1)	5/19 (26.3)	24/102 (23.5)
Sheep	1/4 (25)	0/2 (0)	0/2 (0)	0/2 (0)	1/10 (10)
Pork	2/5 (40)	0/0 (0)	2/4 (50)	0/5 (0)	4/14 (28.6)
Linezolid					
Beef	1/35 (2.9)	3/20 (15)	1/28 (3.6)	4/19 (21.1)	9/102 (8.8)
Sheep	0/4 (0)	0/2 (0)	0/2 (0)	0/2 (0)	0/10 (0)
Pork	1/5 (20)	0/0 (0)	1/4 (25)	0/5 (00	2/14 (14.3)

*np*: number positive isolates, *n*: number of collected isolates.

**Table 3:** Multidrug resistance profiles of *Staphylococcus aureus* isolates to the tested.

Isolates No.	Isolate Codes	Source	Resistance Pattern
1	M13082	Milk	CAZ-OXA-TET-MIN-PEN-G-CAM
2	S9B	Sheep	OXA-PEN-G-ERY
3	A12	Beef	DOXY-OXA-TET-CLI-PEN-G-TMP-TMXERY
4	PD1-Q	Pork	DOXY-OXA-TET-CLI-PEN-G-TMP-TMX-ERY
5	CC5-Q	Beef	OXY-PEN-G
6	C8D	Beef	OXY-PEN-G-ERY-GEN-TMP-TMX-CAM-CAZ
7	B25	Beef	CEF-OXA-MIN-PEN-G-GEN-TMP-TMX-ERY
8	PA8-Q	Pork	CAZ-OXY-PEN-G-TMP-TMX
9	C10	Beef	DOXY-CAZ-AK-OXY-TET-PEN-G- ER
10	PD5-Q	Pork	DOXY-OXA-PEN-G-ER-TET-GEN-TMP-TMX
11	S1A	Sheep	DOXY-OXA-MIN-PEN-G-GEN
12	A3-Q	Beef	DOXY-LZD-TET-PEN-G-CLI-TMP-TMX-ER
13	PA6-Q	Pork	DOXY-LZD-CAZ-OXA-TET-MIN-PEN-G- CLI-ER
14	7B	Beef	OXA-PEN-G-TET-ERY-GEN-CLI-CAM
15	8A-Q	Beef	DOXY, TET, ERY
16	D6-Q	Beef	DOXY-LZD-CAZ-TMP-TMX-ERY
17	M8158	Milk	CIP-DOXY-CAZ-TET-MIN-PEN-G-GEN-TMP-TMX
18	D12	Beef	OXA-PEN-GEN-CAM-TET

CIP: Ciprofloxacin, DOXY: Doxycycline, LZD: Linezoid, CAZ: Ceftazidime, AMK: Amikacin, OXA: Oxacillin, TET: Tetracycline, MIN: Minocycline, CAM: Chloramphenicol, PEN-G: Penicillin-G, CLI: Clindamycin, AMX: Amoxicillin, GEN: Gentamicin, TMP-SMX: Trimethoprim-sulfamethoxazole, ERY: Erythromycin.

**Table 4:** Resistant genes detected from *Staphylococcus aureus* isolated from the Eastern Cape Province.

Genes	Beef *n* (%)	Sheep *n* (%)	Pork *n* (%)	Milk *n* (%)	Total *n* (%)
*bla*Z	9 (69.2)	0 (0)	3 (23.1)	1 (7.7)	13 (100)
*mec*A	1 (100)	0 (0)	0 (0)	0 (0)	1 (100)
*tet*K	20 (76.9)	1 (3.8)	2 (7.7)	3 (11.5)	26 (100)
*tetM*	23 (79.3)	2 (6.9)	4 (13.8)	0 (0)	29 (100)
*msrA*	25 (80)	0 (0)	4 (12.9)	3 (9.7)	31 (100)
*ant (4′)-Ia*	3 (60)	0 (0)	0 (0)	0 (0)	0 (0)
*aph (3′)-1-IIIa*	2 (40)	0 (0)	0 (0)	0 (0)	0 (0)
